# Synovial *CXCL3*^+^*FOSL2*^+^ Macrophages Mediate Inflammation via *FOSL2*/AP-1 in Rheumatoid Arthritis: A Single-Cell Transcriptome Analysis

**DOI:** 10.3390/ijms26199718

**Published:** 2025-10-06

**Authors:** Yiwei Wu, Jinming Yang, Mengke Chen, Xiaoxiang Chen, Shan Cao

**Affiliations:** 1 Department of Rheumatology, Renji Hospital, Shanghai Jiao Tong University School of Medicine, Shanghai 200001, China; wywdoct@163.com (Y.W.); jamieyang1997@163.com (J.Y.); chenmengke9889@gmail.com (M.C.); 2 Department of Allergy, Renji Hospital, Shanghai Jiao Tong University School of Medicine, Shanghai 200001, China

**Keywords:** rheumatoid arthritis, macrophage, AP-1, *FOSL2*, single-cell transcriptome

## Abstract

Macrophages play a central role in joint inflammation and bone destruction in rheumatoid arthritis (RA). While activator protein-1 (AP-1) transcription factors have been implicated in RA pathogenesis, the specific roles of individual AP-1 members in regulating synovial macrophages remain unclear. To address this, two public single-cell transcriptomic datasets were first analyzed to profile synovial macrophages, and then to identify AP-1 family members and associated pathways via differential expression and gene set enrichment analyses. *JUND*, *FOSL2*, and *FOSB* were found to be highly enriched in the RA synovium, and a distinct *CXCL3*^+^*FOSL2*^+^ macrophage subset was identified, characterized by pro-inflammatory, metabolic, and differentiation-related pathways. Intercellular communication analysis further revealed that this *CXCL3*^+^*FOSL2*^+^ macrophage subset interacted with *ACKR1*^+^ endothelial cells within the synovial microenvironment. Validation in a large-cohort bulk transcriptomic dataset, together with functional assays using in vitro *FOSL2* knockdown in U937 cell lines, further confirmed *FOSL2*’s role in promoting macrophage-driven inflammation. Collectively, these findings indicate that *CXCL3*^+^*FOSL2*^+^ macrophages drive RA synovitis via the *FOSL2*/AP-1 axis, highlighting a potential therapeutic target.

## 1. Introduction

Rheumatoid arthritis (RA) is a chronic inflammatory polyarthritis with a global incidence of approximately 1% [1]. It is characterized by immune dysregulation and pannus formation in the synovial microenvironment, leading to articular erosion, joint destruction, and extra-articular manifestations. The resulting joint deformities, functional disabilities, and workforce loss place a significant burden on both individuals and society [2]. Although targeted therapies have been developed against key pathogenic pathways—including the Janus kinase (JAK)-signal transducers and activators of transcription (STAT) signaling [3,4], tumor-necrosis factor-alpha (TNF-α) [5], interleukin (IL)-6 [6], and T cell co-stimulation [7]—a substantial proportion of patients presented inadequate responses or disease relapse during remission. These limitations underscore the need to further explore novel mechanisms and therapeutic targets.

One of the pathological features of RA synovitis is immune cell infiltration, involving both innate and adaptive immune populations [8]. Here, macrophages play a key role in the inflammation of the RA synovium [9,10]. Previous studies have shown that the activation and polarization of macrophages (M1/M2 pattern) are the vital factors in disrupting immune homeostasis and promoting articular inflammation, accompanied by the release of pro-inflammatory cytokines and chemokines, T cell activation, and remodeling by the extracellular matrix formation [11,12]. Recent single-cell analyses have revealed significant diversity among synovial macrophages, identifying distinct subsets defined by specific functional markers. For example, MerTK^+^CD206^+^ and MerTK^+^TREM2^high^ macrophages are associated with efferocytosis and tissue repair, while macrophages in anti-citrullinated protein antibody-negative RA show increased expression of CCL13, CCL18, and MMP3, reflecting their roles in chemotaxis and matrix remodeling [8,13,14,15]. The findings suggest the synovial macrophage components may be more complex and multi-functional than previously recognized.

The complex functions and components of synovial macrophages are now acknowledged as a consequence of transcriptional regulation, environmental factors, and epigenetic induction, but the underlying mechanism is still unclear. The activator protein 1 (AP-1) family, a group of transcription factors involved in inflammation, proliferation, and apoptosis, has been implicated in RA pathogenesis. AP-1 consists of homo- and heterodimeric complexes formed by FOS and JUN proteins [16]. FOS proteins consist of FRA1 (coded by *FOSL1*), FRA2 (coded by *FOSL2*), FOSB (coded by *FOSB*), and c-FOS (coded by *FOS*), while the JUN family includes JUNB, JUN, and JUND [17]. FRA1 has been reported to be associated with the active phase of RA and to promote inflammatory responses in synovial macrophages [18]. Moreover, the higher expression of JUN with FRA1 can be observed in arthritic models and can be inhibited by a cyclin-dependent kinase inhibitor, suggesting the promotion of cellular growth by the AP-1 family [19]. However, it is unclear how the rest of the AP-1 family regulates the synovial macrophages in RA.

In the study, we integrated single-cell RNA sequencing (scRNA-seq) and bulk RNA-seq to construct a comprehensive cellular and transcriptomic landscape in the RA synovium, with a particular focus on macrophage heterogeneity at two aspects, primarily, the intrinsic functions, and secondly, the cellular interaction. We identified a distinct subset, *CXCL3*^+^*FOSL2*^+^ macrophage, which exhibited multifunctional properties and interacted with *ACKR1*^+^ endothelial cells. This specialized population reflects an immunophenotypic divergence within the synovial macrophage compartment and contributes to the inflammatory milieu of RA.

## 2. Results

### 2.1. Single-Cell Landscape of Synovial Tissue in RA

**Figure 1:**
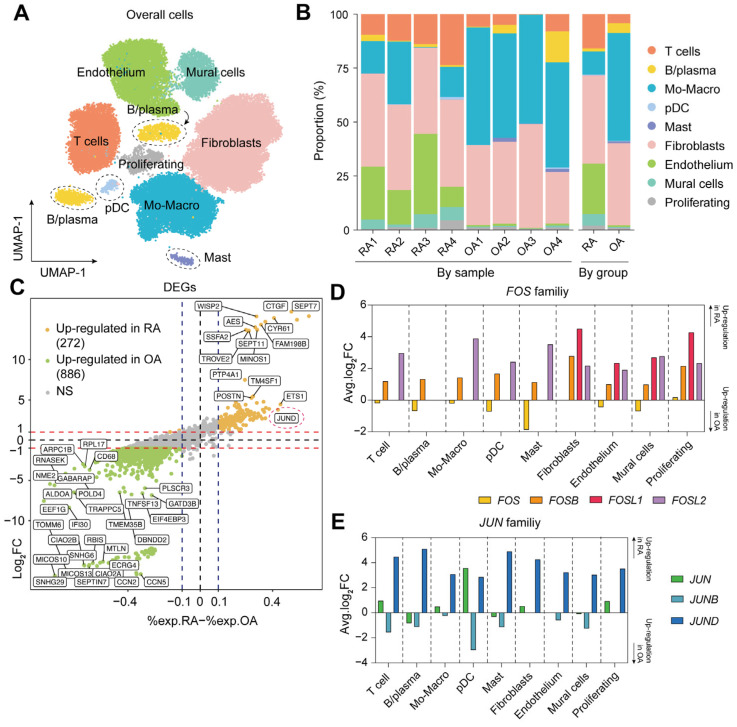
The single-cell landscape and gene expression profiles of the RA and OA synovium. (**A**) UMAP visualization of 47,672 cells from eight patients, colored by cell types. (**B**) Stacked bar chart for the proportion of cell types split by sample or by group. (**C**) Diagonal volcano plot for DEGs between RA and OA. (**D**) The expression of the *FOS* families by cell types between RA and OA. (**E**) The expression of the *JUN* families by cell types between RA and OA.

The scRNA-seq datasets of synovial tissues from RA (GSE200815) and OA (GSE248455) were combined to depict a single-cell landscape of the synovium. Based on the canonical markers (Figure S1A), a total of 47,672 high-quality single cells were clustered into nine distinct cell types, including T cells, B/plasma cells, monocyte-macrophage lineage, pDCs, mast cells, fibroblasts, ECs, mural cells, and proliferating cells (Figure 1A). Specifically, compared to OA, the proportions of T cells, endothelium, and mural cells were relatively higher in RA, while the proportion of monocyte-macrophage lineage of the RA group was lower than that of the OA group (Figure 1B and Figure S1B). No statistically significant differences were observed in B/plasma cells, pDCs, mast cells, fibroblasts, and proliferating cells between the two groups (Figure S1B). These results reflected a distinct immune microenvironment in the RA synovium, including T cell infiltration, pannus formation, and macrophage remodeling.

### 2.2. Elevated Expression of FOSL2 and JUND in RA Synovium

To further detect the AP-1 family, we performed differential gene expression analysis using the FindMarkers function. A sum of 272 up-regulated genes were expressed in the RA synovium, including *JUND*, *FOSL2* and *FOSB* (*JUND*, avg.log_2_FC = 3.78, *P*_adj_ < 0.001; *FOSL2*, avg.log_2_FC = 3.21, *P*_adj_ < 0.001; *FOSB*, avg.log_2_FC = 1.98, *P*_adj_ < 0.001) (Figure 1C and Table 1). However, *JUN* showed no significant difference in expression between the RA synovium and the OA synovium (avg.log_2_FC = 0.41) (Table 1). *FOS*, *FOSL1*, and *JUNB* were not reported owing to their low expression, less than 25% in either the whole RA tissues or OA ones.

Regarding the AP-1 family potentially differing among different cellular clusters, we applied the group-to-group comparisons by cell types. As shown in Figure 1D,E, *JUND*, *FOSL2*, and *FOSB* were consistently highly expressed in all cell types in RA synovium compared to those of OA, while *FOSL1* was up-regulated only among fibroblasts, endothelium, mural cells, and proliferating cells. Interestingly, the expression of *FOSL2* by RA monocyte-macrophage lineage was more remarkable; *FOSL2*-related pathways were also highly enriched in RA synovial macrophages, involving cell development, inflammation, and metabolism (Figure S2A,B).

### 2.3. Immunophenotype Divergence of Macrophages in RA

**Figure 2:**
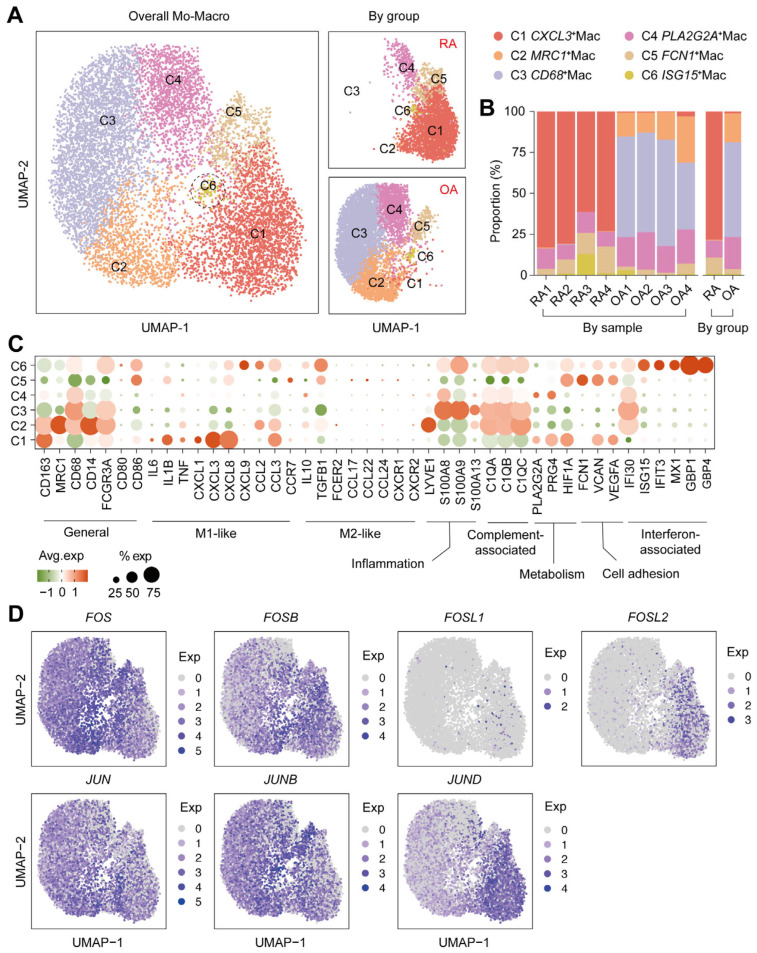
The monocyte-macrophage subclusters and the AP-1 expression. (**A**) Sub-clustering division of monocyte-macrophage lineage into six sub-clusters and split by group. (**B**) Stacked bar chart for the proportion of macrophage sub-clusters split by sample or by group. (**C**) Functional genes expression by macrophage sub-clusters. (**D**) Regional visualization for the whole AP-1 family expressed by monocyte-macrophage lineage.

Since monocyte-macrophage lineage is closely associated with activity of RA synovitis [20], we further focused on the macrophage constitution and its expression of the AP-1 family. Thus, we selected the monocyte-macrophage lineage and further subdivided it into six sub-clusters, including *CXCL3*^+^Mac (C1), *MRC1*^+^Mac (C2), *CD68*^+^Mac (C3), *PLA2G2A*^+^Mac (C4), *FCN1*^+^Mac (C5), and *ISG15*^+^Mac (C6) (Figure 2A). Meanwhile, it showed significant divergence between RA and OA; specifically, *CXCL3*^+^Mac (C1) was the predominant cluster in the RA synovium, while OA was characterized by *MRC1*^+^Mac (C2) and *CD68*^+^Mac (C3) (Figure 2A,B). As for the functional genes, *CXCL3*^+^Mac (C1) highly expressed some of the M1-like markers (*IL1B*, *IL6*, *TNF*, *CXCL1*, *CXCL3*, *CXCL8*, and *CCL3*), together with metabolism-associated (*PLA2G2A*, *PRG4*, and *HIF1A*) and intercellular adhesion (*VCAN* and *VEGFA*) genes, which suggested that *CXCL3*^+^Mac had similar features to pro-inflammatory M1 macrophages. Whereas *MRC1*^+^Mac (C2) and *CD68*^+^Mac (C3), the predominant cluster of the OA synovium, mainly expressed the complement-associated genes (*C1QA*, *C1QB*, *C1QC*) and *IFI30*, an interferon-related gene (Figure 2C).

With regard to the expression of AP-1 members, *FOSL2* was significantly expressed by the predominant cluster (C1) of RA, and *JUND* was mainly expressed in C1 and a minority of other clusters. However, *FOS*, *FOSB*, *JUN*, and *JUNB* were widely expressed in all macrophages, and *FOSL1* was only expressed among a very small number of cells (Figure 2D).

To sum up, these findings indicate a significant immunophenotypic and functional divergence in the RA synovium, closely associated with elevated *FOSL2* expression, particularly in the prominent *CXCL3*^+^Mac (C1) subset.

### 2.4. Multiple Functions of Synovial CXCL3^+^FOSL2^+^Mac on RA

**Figure 3:**
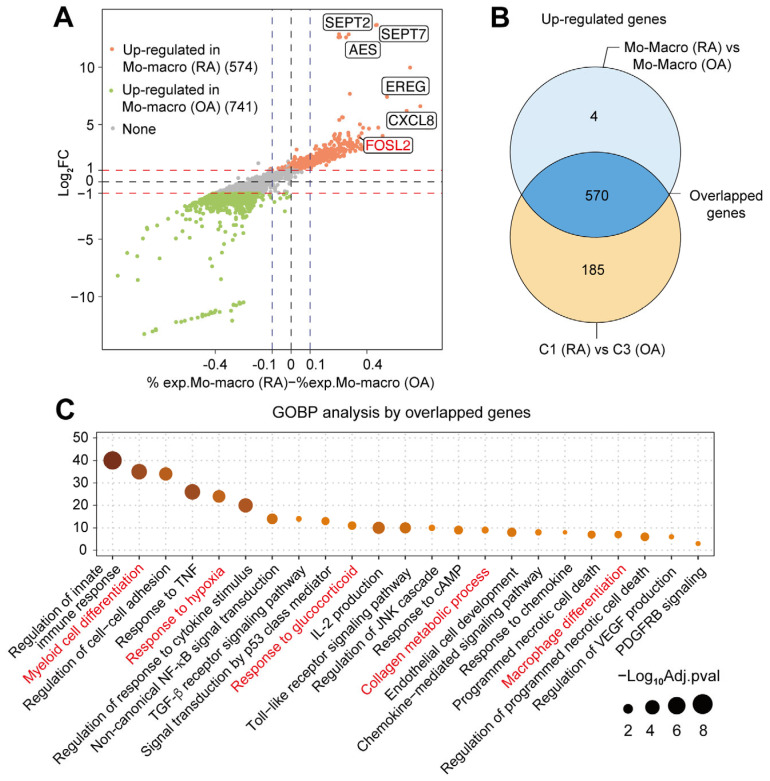
The pathway enrichment of RA synovium. (**A**) Diagonal volcano plot for DEGs between RA and OA synovial macrophages. (**B**) Venn diagram showing the overlap between up-regulated DEGs of RA vs. OA macrophages (574) and up-regulated DEGs of C1 (RA) vs. C3 (OA) (755), with 570 overlapping genes. (**C**) The gene set enrichment of overlapped DEGs on GOBP pathways, with *FOSL2*-related ones colored in red. Abbreviations: cAMP, cyclic adenosine monophosphate; PDGF, platelet-derived growth factor; TGF-β, transforming growth factor-beta; TNF, tumor-necrosis factor; JNK, c-Jun N-terminal kinase; VEGF, vascular endothelial growth factor.

To quantify the contribution of the synovial C1 cluster to the overall transcriptional differences between RA and OA macrophages (as shown in Figure 3A), we compared C1 with the predominant OA cluster (C3). There were 755 up-regulated DEGs in C1, of which 570 overlapped with the 574 up-regulated DEGs identified between RA and OA macrophages, yielding an overlap rate of 99.30% (=570/574) (Figure 3B), suggesting that a large proportion of functional genes contributing to the overall genes of synovial RA macrophages were mainly from *CXCL3*^+^*FOSL2*^+^Mac (C1). These 570 overlapped genes were mainly enriched in the pathways related to inflammation, immunity, metabolism, tissue remodeling, and myeloid cell differentiation and development, with five of these pathways involving *FOSL2* (Figure 3C). The enrichment of specific pathways suggests that *CXCL3*^+^*FOSL2*^+^Mac (C1) might contribute to the onset and progression of RA synovitis through multiple pathogenic mechanisms. Then, we merged the 570 up-regulated genes and defined them as the ‘*CXCL3*^+^*FOSL2*^+^Mac signature’ to represent the multiple functions of pro-inflammation, active metabolism, and cell differentiation of *CXCL3*^+^*FOSL2*^+^Mac.

### 2.5. CXCL3^+^FOSL2^+^Mac Signature Enrichment by a Bulk Level Validation

**Figure 4:**
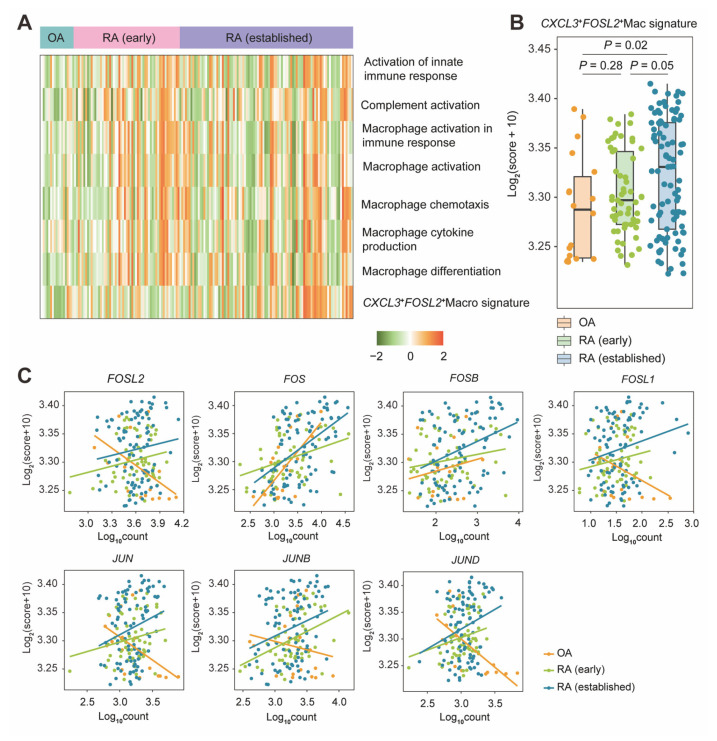
The validation of bulk RNA-seq on *FOSL2*^+^Mac signature. (**A**) The enrichment of macrophage-associated pathways among the patients with OA, early RA, and established RA. (**B**) The enrichment and comparison of *FOSL2*^+^Mac signature among OA, early RA, and established RA; statistical significance was assessed using the Kruskal–Wallis H test. (**C**) The association between the enrichment of the *FOSL2*^+^Mac signature and the expression of the AP-1 family.

To further validate the pathway enrichment in a large sample and at a bulk level, the *CXCL3*^+^*FOSL2*^+^Mac signature and seven macrophage function-related pathways were applied on bulk counts of synovial tissues (GSE89408 in Table 2). Alongside macrophage function-related pathways, the *CXCL3*^+^*FOSL2*^+^Mac signature was more enriched in RA compared to OA (Figure 4A). Moreover, the enrichment score of the *CXCL3*^+^*FOSL2*^+^Mac signature in established RA was higher than those of RA in early status or OA (Figure 4B).

As for each AP-1 member, with the increasing expression count of *FOSL2*, *FOSL1*, *JUN*, *JUNB*, and *JUND*, the enrichment of the *CXCL3*^+^*FOSL2*^+^Mac signature was generally elevated in early RA and established RA, and OA conversely showed a negative association. But these three groups presented the same trend in the signature score when *FOS* and *FOSB* increased (Figure 4C). To sum up, the results suggest that the *CXCL3*^+^*FOSL2*^+^Mac signature was associated with the RA synovium of the late phase.

### 2.6. Validation by M1/M2 Pattern In Vitro

**Figure 5:**
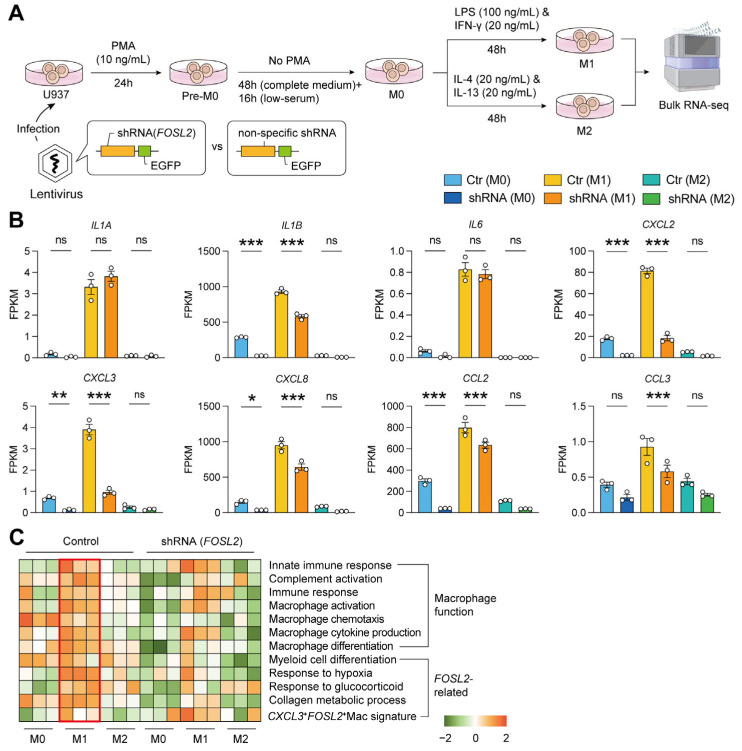
M1/M2 polarization in vitro after knockdown of *FOSL2*. (**A**) The workflow of M1/M2 polarization in vitro using U937 cells infected by shRNA-*FOSL2*-containing lentivirus; (**B**) The comparisons of M1 markers after knockdown of *FOSL2*; Statistical significance was determined by one-way ANOVA, *, *p* < 0.05; **, *p* < 0.01; ***, *p* < 0.001; ns, non-significant. (**C**) The gene set enrichment among shRNA (M0/M1/M2) and control groups, with the red box highlighting the M1 cells without *FOSL2* knockdown as the control.

The above single-cell profile has shown that *CXCL3*^+^*FOSL2*^+^Mac was more similar to the pro-inflammatory macrophages (M1). Then, we knocked down *FOSL2* in the U937 cell line and conducted macrophage polarization to explore the function of *FOSL2* on the macrophages (Figure 5A). After the confirmation of M1/M2 phenotype by their characteristic markers (Figures S3 and S4) [21], we found that the expression of several M1 markers (*IL1B*, *CXCL2*, *CXCL3*, *CXCL8*, *CCL2*, and *CCL3*) significantly decreased in the M1 cells with the knockdown of *FOSL2*, while M2 showed a stably lower expression of these markers whether in non-treatment or knockdown of *FOSL2* (Figure 5B). Within the M2 cells, the expression of some M2 markers (*TGFB1*, *CXCR1*, and *CXCR2*) similarly reduced but to a relatively small extent (Figure S5). In addition, after the knockdown of *FOSL2*, the expression of *FOS* and *FOSB* collaboratively decreased in the M1 (Figure S6).

To explore how the *FOSL2*-related pathways mentioned in Figure 3C changed after the knockdown of *FOSL2*, we compared the enrichment of functional gene sets among M0/M1/M2 cells with or without the knockdown of *FOSL2*. As shown in Figure 5C, the enrichment of certain *FOSL2*-related pathways (including myeloid cell differentiation, hypoxia response, and response to glucocorticoid) decreased in M1 cells alongside the knockdown of *FOSL2*, while most *FOSL2*-related pathways, as well as macrophage functions, were reserved or enriched in the M1 cells without *FOSL2* knockdown. These results suggested that *FOSL2* was closely associated with maintaining multiple functions of M1 cells.

### 2.7. CXCL3^+^FOSL2^+^Mac Interacted with ACKR1^+^Endothelium

**Figure 6:**
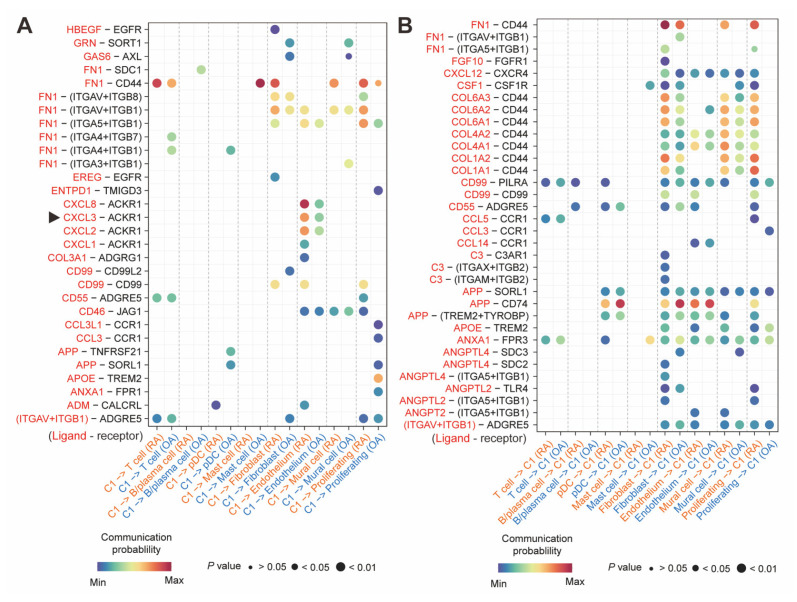
The intercellular communications of *CXCL3*^+^*FOSL2*^+^Mac (C1). (**A**) The communication with C1 as the signal transmitter. The arrows indicated *CXCL3*^+^*FOSL2*^+^Mac communicated with ECs via *CXCL3*-*ACKR1* pair. (**B**) The communication with C1 as the signal receiver.

Apart from the intrinsic functions and signaling analysis, it was found that there were cellular interactions among *CXCL3*^+^*FOSL2*^+^Mac and other cell types in the inflammatory microenvironment. As shown in Figure 6A, compared to the OA, *CXCL3*^+^*FOSL2*^+^Mac (C1) of RA were more likely to interact with ECs via *CXCL2*, *CXCL3*, and *CXCL8* as the ligands, and *ACKR1* served as the receptors.

Conversely, when *CXCL3*^+^*FOSL2*^+^Mac (C1) served as the communication receivers, they exhibited enhanced ECM-receptor signaling from ECs, primarily involving communication pairs between collagen-related genes and *CD44*, such as *COL4A1*-*CD44* and *COL4A2*-*CD44* (Figure 6B). This mutual communication between *CXCL3*^+^*FOSL2*^+^Mac (C1) and ECs constituted the inflammatory microenvironment in the RA synovium. In addition, the ECM-receptor signaling involving collagen-related genes and *CD44* was also manifested in the communications between *CXCL3*^+^*FOSL2*^+^Mac (C1) and fibroblasts, as well as mural cells and proliferating cells.

## 3. Discussion

In this study, we constructed a single-cell landscape of AP-1 expression across synovial cells from RA and observed that several AP-1 family members (including *FOSL2*, *FOSB*, and *JUND*) were highly enriched in the inflamed RA synovium. Of the synovial cells, we identified a distinct macrophage cluster characterized by elevated expression of *CXCL3* and *FOSL2*, as well as several signatures related to M1-like pro-inflammation, active metabolism, and cell differentiation, further validated by bulk RNA-seq of a large-sample cohort. In vitro validation with *FOSL2* knockdown in the monocyte cell line further demonstrated that the proinflammation by macrophages could be regulated by *FOSL2*. Moreover, this *CXCL3*^+^*FOSL2*^+^macrophage subset also exhibited interactions with *ACKR1*^+^ECs, which might facilitate the inflammation in the synovial microenvironment. These results suggest that synovial *CXCL3*^+^*FOSL2*^+^macrophages may be related to the inflammation in RA.

Consistent with the previous studies [22,23], a higher proportion of T cells, endothelial cells, and mural cells was observed in the RA synovium, reflecting T cell infiltration and pannus formation as characteristic pathological manifestations of RA. Interestingly, in spite of a relatively lower proportion of RA monocyte-macrophage lineage than that of OA, there remained an immunophenotypic divergence and macrophage heterogeneity within RA synovium; in particular, the pathways related to cell development, inflammation, and metabolism were significantly enriched in RA synovial macrophages. Similarly, Zhang et al. [14] found that pro-inflammatory phenotypes apparently occurred in the RA monocyte-macrophage lineage, even though there was no significant difference in monocyte counts between RA and OA. The study of Zheng [24] also showed an immunophenotypical bias toward pro-inflammatory macrophage subsets in active RA synovitis. Thus, there may exist an immunophenotypic divergence and macrophage diversity within the RA synovium, which suggests underlying special intrinsic gene expression profiles in RA synovial macrophages.

Indeed, the macrophages between RA and OA showed a significant difference in the gene expression profiles, especially some AP-1 family members, including *FOSL2*, *FOSB*, and *JUND*. Within the macrophage sub-clusters, *FOSL2* and *JUND*—rather than *FOSL1* or other AP-1 family members—were the predominant genes for distinguishing between RA and OA. Inconsistent with our study, Hannemann et al. [18] found that FRA1 (*FOSL1*) was an important transcription factor regulating metabolic activity in synovial macrophages among active RA. In contrast, our data demonstrated a higher expression of *FOSL1* in fibroblasts and vasculatures but a sparse expression in certain macrophage sub-clusters in the RA synovium; while *FOSL2* was predominantly expressed in the RA synovium, particularly in the distinct macrophage sub-cluster, *CXCL3*^+^Mac (C1). This subset highly expressed several M1 markers—demonstrating their potential for promoting inflammation—as well as *FOSL2*-related pathways related to immunity, metabolism, tissue remodeling, and myeloid cell differentiation. Previous studies have revealed that synovial macrophages are involved in pro-inflammation by cytokines or cell–cell interactions [15,25,26], metabolism regulations [27,28], differentiation [29], etc., but few have specifically linked *FOSL2* to the functions of macrophages. Our findings emphasize that *FOSL2* distinctively mediates the multiple pathogenic mechanisms of the synovial *CXCL3*^+^macrophage.

Meanwhile, the above results were further validated by the bulk RNA-seq transcriptome. In the large-sample cohort analysis, the gene signature linked to *CXCL3*^+^*FOSL2*^+^macrophages was closely associated with progressed RA synovitis, but not cases in an early-stage or healthy status. On the other hand, the pro-inflammatory activity of the *CXCL3*^+^*FOSL2*^+^macrophage subset was also confirmed to be related to *FOSL2* by the experiments in vitro. We observed that expression of several M1 markers significantly decreased with *FOSL2* knocked down. Gao and colleagues [30] similarly found that the inflammation by macrophages could be inhibited by FRA2 (*FOSL2*)-targeting medicine, and the latter had a therapeutic effect for RA-mimicking animal models. These findings support the notion that RA inflammation is primarily promoted by the M1-like pro-inflammation and multifunctional activity of the *CXCL3*^+^*FOSL2*^+^macrophage subset, which could be driven by *FOSL2*. Further experiments are needed to elucidate the precise mechanisms.

In addition to the intrinsic multifunction mediated by *FOSL2*, the *CXCL3*^+^*FOSL2*^+^Mac subset also exhibited potential interactions with the *ACKR1*^+^endothelium, with *CXCL3* as the ligand. *ACKR1* has been reported as a distinctive marker for ECs, especially the postcapillary veins [31], suggesting that the interaction between *CXCL3*^+^*FOSL2*^+^Mac and the *ACKR1*^+^endothelium concentrates in the postcapillary networks. This condition might facilitate the migration and spread of macrophages. A study by Girbl et al. [32] demonstrated that certain myeloid cells, such as neutrophils, relied on the conjunction between CXCL2 and ACKR1 to transmigrate through the vascular networks. Another study also found that rheumatoid synovial ECs could promote the trans-endothelial migration of monocytes [33]. Based on this, we hypothesize that synovial *CXCL3*^+^*FOSL2*^+^macrophages may similarly utilize the CXCL3-to-ACKR1 conjunction to migrate within the vascular pannus, thereby amplifying inflammation.

In addition, *CXCL3*^+^*FOSL2*^+^Mac and fibroblasts also presented strong communications in the RA synovitis. This close relationship mainly relied on the collagen-related ECM-receptor signaling. Previous studies have demonstrated that fibroblast-like synoviocyte (FLS) is a key effector of cells in RA synovitis and drives ECM remodeling via cytokines and proteases [34,35]. ECM formation by the fibroblasts might provide structural support for the adhesion and residence of the macrophages by CD44 [36]; meanwhile, some cytokines (such as tenascin-C, which activates TLR4 and sustains joint inflammation [37], and biglycan, which triggers TLR2/TLR4 signaling and TNF production [38]) presenting in the ECM might also promote the pro-inflammation of the macrophages. Thus, the relationship between *CXCL3*^+^*FOSL2*^+^Mac and fibroblasts, communicating via ECM-receptor signaling, is another possibility for RA synovitis.

There are some limitations in our study. First, healthy synovial tissues were not available due to the clinical restrictions in collecting samples from healthy donors and the unavailability of the shared data. As an alternative, we utilized synovial tissues from OA patients with mild knee pain, which are generally considered to present a low-inflammatory baseline. Second, our study primarily relied on bioinformatic analyses integrating scRNA-seq and bulk RNA-seq transcriptomic data. While these approaches provided valuable insights, further validation by multi-omics and animal experiments will be warranted to elucidate the underlying mechanisms via *FOSL2* and the spatiotemporal dynamics of RA progression.

## 4. Materials and Methods

### 4.1. Single-Cell Transcriptome Data Processing

A public scRNA-seq dataset of synovial tissues was obtained, which included four patients with active RA under the accession number GSE200815 from the Gene Expression Omnibus (GEO) database [39]. Owing to the unavailability of synovial tissues of healthy donors, we selected four patients with osteoarthritis (OA) experiencing mild knee pain as the comparisons (GSE248455) [40] (Table 2).

Raw sequence data of these datasets were processed into gene expression matrices using Cellranger [v 8.0.1, 10x Genomics (Pleasanton, CA, USA)], with the human GRCh38 genome as the mapping reference. The gene expression matrices were analyzed using the Seurat R package (v5.2.1). The cells with a number of detected features between 200 and 5000 and a percentage of mitochondrial genes below 20% were retained; ultimately, a total number of 47,672 high-quality singlets were included. Normalization was performed to reduce the potential variance among all samples, and 5000 highly variable genes were generated. We scaled and dimensionally reduced the data by principal component analysis using the generated variable genes. Batch effects were minimized using the Harmony R package (v1.2.3). The top 50 significant principal components were selected for uniform manifold approximation and projection (UMAP), and the neighboring clusters were found by 50 principal components and a resolution of 0.3.

### 4.2. Cell Annotation

The FindMarkers function was used to compare each cluster to the resting state to find the differentially expressed genes (DEGs). We used the following canonical markers to identify the cell types: *CD3E*, *CD3D*, and *CD3G* for T cells; *MS4A1*, *CD79A*, and *JCHAIN* for B/plasma cells; *CD163*, *MRC1*, *CD68*, and *CD14* for the monocyte-macrophage lineage; *LILRA4*, *CLEC4C*, and *IL3RA* for plasmacytoid dendritic cells (pDCs); *MS4A2* and *CPA3* for mast cells; *PDGFRL*, *DCN*, and *PRG4* for fibroblasts; *VWF* and *SELE* for endothelial cells (ECs); *MYH11*, *ACTA2* for mural cells; and *MKI67* and *TOP2A* for proliferating cells.

### 4.3. Differentially Expressed Gene Detection

DEGs were detected using the FindMarkers function of the Seurat R package (v5.2.1), and the *p* values were calculated through the Wilcoxon rank sum test and adjusted using the Bonferroni correction. Logarithmical fold changes (FCs) of expression with two as the base for each gene showed the difference in gene expression of the specified groups compared to the comparisons, and each gene had an expression percentage in both the specified groups of cellular types and the compared ones. Genes were taken into the calculation when they were expressed in at least 25% of the dominant groups. Those genes with adjusted *p*-values of less than 0.01, average Log_2_FC of more than 1, and a difference in expression percentage of more than 0.1 were defined as the positive DEGs.

### 4.4. Gene Set Enrichment Analysis

The gene set enrichment analysis (GSEA) was performed using gseGO and gseKEGG functions [clusterProfiler R package (v4.10.1)] on the pathways from Biological Process of Gene Ontology (GOBP) and Kyoto Encyclopedia of Genes and Genomes (KEGG), respectively. Here, a list of genes was ranked according to their expression (namely, avg.log_2_FC), and the enrichment score would be calculated through the mapping of the gene sets onto the gene lists. The pathways inclined to enrich in RA if the values of the enrichment score were more than 1.0, while the values less than −1.0 showed that the pathways were enriched in OA. *p* values adjusted by Benjamini–Hochberg correction were considered statistically significant when they were less than 0.05.

### 4.5. GOBP Analysis

The DEGs for the clusters of interest were enriched using GOBP analysis, a way of analyzing the biological process of the genes to explore the functional genes. Here, we used the clusterProfiler (v4.10.1) based on the enrichGO function. *p* values were adjusted by the Benjamini–Hochberg correction.

### 4.6. Intercellular Communication

The intercellular communications between macrophage sub-clusters and other cell types were analyzed based on the Cellchat (v2.2.0) R package according to ligand-receptor pairs of three aspects, extracellular matrix (ECM)-receptor, secreted signaling, and cell–cell contact. Communication probability served as a measurement for evaluating the probability of communication signaling.

### 4.7. The Knockdown of FOSL2

We used U937 cells, a monocytic cell line, to conduct macrophage polarization in vitro, and knockdown *FOSL2* to verify the function of the gene on macrophages. Briefly, lentiviral vectors carrying shRNA targeting *FOSL2* (targeted sequencing, 5′-GCAGTGAGTATTGGAAGACTT-3′) and a non-specific shRNA control (target sequencing, 5′-CCTAAGGTTAAGTCGCCCTCG-3′) were constructed and provided by OBiO Technology (Shanghai, China). The recombinant vectors and the helper plasmids were co-transfected into 293T cells, and the lentiviral particles were prepared. The U937 cells in the logarithmic growth phase were infected by the lentiviral particles assembled with shRNA-*FOSL2* and non-specific shRNA, with uninfected U937 cells as the control group. These three groups were all planted and amplified at the complete medium (RPMI 1640 supplemented with 10% FBS [Gibco, Thermo Fisher Scientific (Grand Island, NY, USA)] and 1% penicillin-streptomycin [Gibco, Thermo Fisher Scientific (Grand Island, NY, USA)], containing 1 μg/mL puromycin [InvivoGen (Toulouse, France)]). The cells were observed daily until the control group grew at an extremely low speed due to the screening by the puromycin. The shRNA-*FOSL2* and non-specific shRNA groups were confirmed by the presence of green fluorescence and cultured in the puromycin-free medium. The U937 and 293T cell lines were obtained from Cell Bank of the Chinese Academy of Sciences (Shanghai, China).

### 4.8. Macrophage Polarization In Vitro (M1/M2 Pattern)

After the transfection, the transfected U937 cells in the shRNA-*FOSL2* and non-specific shRNA groups were seeded at a density of 1 × 10^6^ cells/mL in complete medium. A quantity of 10 ng/mL phorbol-12-myristate-13-acetate (PMA) [MedChemExpress (Monmouth Junction, NJ, USA)] was added for stimulation for 24 h. After removing PMA, the cells in the pre-M0 status were cultured in PMA-free complete medium and low-serum medium for 48 h and 16 h in succession, after which the cells were induced into M0. M1 cells were induced from M0 by lipopolysaccharides (LPS) (100 ng/mL) [MedChemExpress (Monmouth Junction, NJ, USA)] and interferon-γ (20 ng/mL) [Peprotech (Rocky Hill, NJ, USA)] for 48 h. M2 were induced by IL-4 (20 ng/mL) [Peprotech (Rocky Hill, NJ, USA)] and IL-13 (20 ng/mL) [Peprotech (Rocky Hill, NJ, USA)] for 48 h. M0 treated by the same volume of phosphate-buffered saline (PBS) [Cytiva (Marlborough, MA, USA)] and for the same time was defined as the control group. Each group had three biological replicates.

The mRNAs of the M0/M1/M2 cells were extracted by TRIzol reagent [Invitrogen, Thermo Fisher Scientific (Carlsbad, CA, USA)] and were reverse-transcribed into cDNAs [Takara Bio Inc. (Kusatsu, Shiga, Japan)]. The cDNA libraries were sequenced with an Illumina NovaSeq 6000 (San Diego, CA, USA) with paired-end flow cells. Raw read quality was assessed using FastQC [41]. The raw sequencing data were transformed to fastq.gz format and were mapped into a matrix of expression counts using the human GRCh38 genome as the reference. Then, we used the non-redundant exon lengths as the lengths of the gene to calculate the fragments per kilobase of the transcript per million mapped reads (FPKM), which stand for the abundance level of the interested genes in the samples [42]. The FPKM of selected genes was used for the comparisons between different groups. The matrix of gene counts would be applied to the DEG comparison (by DESeq2 [v1.48.1] R package) and gene set variance analysis (by clusterProfiler [v4.10.1] R package).

### 4.9. The Analysis of Bulk Transcriptome Data

To validate gene expression and gene set enrichment of the scRNA transcriptome, we obtained a large-sample dataset of bulk transcriptome (GSE89408) from GEO [43]. We selected the raw gene counts matrix of synovial tissues from 18 patients diagnosed with OA, 57 with early RA, and 93 with established RA. We applied the *CXCL3*^+^*FOSL2*^+^macrophage signature and several macrophage-associated functional pathways (downloaded from the MsigDB database [44]) to the bulk gene count among three groups, using gene set variance analysis to explore the gene set enrichment at the level of individuals. The association between the enrichment score of specific pathways and the AP-1 count was calculated in order to explore the changed enrichment of the gene sets, along with AP-1 expression.

### 4.10. Statistical Analysis

All statistical analyses were conducted in R (v4.4.0). An independent samples *t*-test or one-way ANOVA was used to compare the difference between groups when the data type was in normal distributions; otherwise, the Mann–Whitney U test or the Kruskal–Wallis H test was used. A two-tailed *p* ≤ 0.05 was considered statistically significant.

**Table 2:** Accessibility of publicly shared datasets of synovial tissues enrolled in this study.

Dataset ID	Sequence Types	Platform	Tissues	Constitution
GSE200815	scRNA-seq (10x Genomics)	Illumina NovaSeq 6000	synovial tissues	Four RA with moderate to high disease activity *
GSE248455	scRNA-seq (10x Genomics)	Illumina NovaSeq 6000	synovial tissues	Four OA with mild knee pain ^†^
GSE89408	Bulk RNA-seq	GPL11154 Illumina HiSeq 2000 (San Diego, CA, USA)	synovial tissues	18 OA, 57 early RA, and 93 established RA ^‡^

* The RA patients naïve of treatment presented increased CRP and ESR levels and high DAS28 scores, suggesting moderate to high disease activity [39]. ^†^ The OA patients with less pain presented advanced structural damage but with mild inflammatory changes. They were mainly treated with common analgesics [40]. ^‡^ Early RA: disease duration ≤ 12 months from diagnosis and DMARD-naïve. Established RA: disease duration > 12 months from diagnosis and prior DMARD or anti–TNF-α treatment [43]. Data-availability note: the original study reports 95 established RA cases, but the GEO gene-count matrix contains 93; therefore, *n* = 93 was used for bulk RNA-seq validation.

## 5. Conclusions

Our study uncovered a transcriptomic landscape of AP-1 expression by RA synovial cells, revealing that *FOSL2*, *JUND*, and *FOSB* were highly enriched in the inflamed synovium of RA. A unique macrophage cluster, *CXCL3*^+^*FOSL2*^+^macrophage, was related to pro-inflammation, active metabolism, and cell differentiation, as well as the interaction with the *ACKR1*^+^endothelium. These results suggested that synovial *CXCL3*^+^*FOSL2*^+^macrophage could mediate the immunophenotype divergence of RA synovitis. The findings provide insight into the understanding of the function of *CXCL3*^+^*FOSL2*^+^macrophages in how RA synovitis develops, which not only enhances our understanding of the cellular and transcriptional mechanisms underlying RA but also highlights *FOSL2* as a potential therapeutic target for relieving synovial inflammation.

## Figures and Tables

**Table 1:** The expression of AP-1 members in RA synovium compared to OA.

Genes	Avg.log_2_FC *	% exp in RA ^†^	% exp in OA ^‡^	Adjusted *p* Values ^§^
*JUND*	3.78	0.907	0.477	0
*FOSL2*	3.21	0.427	0.158	0
*FOSB*	1.98	0.792	0.668	0
*JUN*	0.41	0.753	0.805	6.5 × 10^−52^

* Avg.log_2_FC indicates the average expression of the genes in the RA synovium compared to the OA synovium, which was displayed as logarithmic fold change with 2 as the base. ^†^ Item indicates the proportion of cells expressing the interested gene in RA synovium. ^‡^ Item indicates the proportion of cells expressing the relevant gene in the OA synovium. ^§^ Wilcoxon rank-sum test; Bonferroni adjusted *p* values.

## Data Availability

The data analyzed in this study were mainly derived from publicly available resources: two single-cell RNA-seq datasets and one bulk RNA-seq dataset downloaded from the GEO database (accession numbers: GSE200815, GSE248455, and GSE89408). In addition, the bulk RNA-seq dataset generated from polarized U937 cells in this study has been deposited in the SRA database (accession number: PRJNA1309597).

## Supplementary Materials

The following supporting information can be downloaded at https://www.mdpi.com/article/10.3390/ijms26199718/s1.

## Abbreviations

The following abbreviations are used in this manuscript:

AP-1	activator protein-1
DEG	differentially expressed gene
EC	endothelial cell
ECM	extracellular matrix
FC	fold change
FPKM	fragments per kilobase of transcript per million mapped reads
GEO	Gene Expression Omnibus
GOBP	Biological Process of Gene Ontology
GSEA	gene set enrichment analysis
IL	interleukin
JAK	Janus kinase
KEGG	Kyoto Encyclopedia of Genes and Genomes
OA	osteoarthritis
pDC	plasmacytoid dendritic cell
RA	rheumatoid arthritis
SMAD	suppressor of mother against decapentaplegic
STAT	signal transducers and activators of transcription
TNF	tumor-necrosis factor
UMAP	uniform manifold approximation and projection
