# Supplementary material for: Synovial CXCL3+FOSL2+ Macrophages Mediate Inflammation via FOSL2/AP-1 in Rheumatoid Arthritis: A Single-Cell Transcriptome Analysis
Source: Int J Mol Sci. 2025 Oct 6;26(19):9718. doi: 10.3390/ijms26199718 (PMC12524658; doi:10.3390/ijms26199718)
Supplement: Supplementary file 1 [file ijms-26-09718-s001.zip › Supplementary files.pdf]

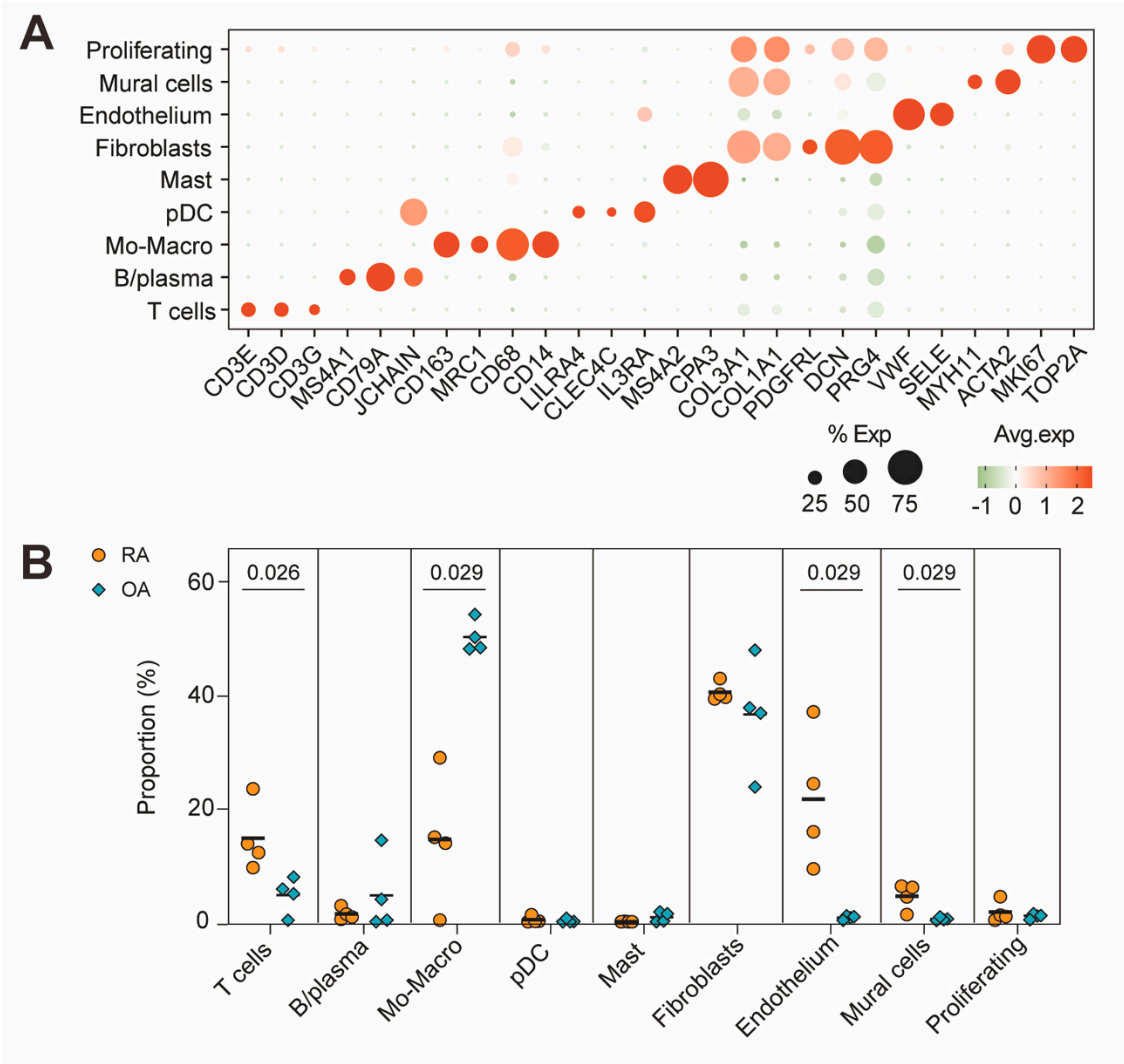

Figure S1. The canonical markers and proportional comparison of RA and OA synovium. A. Canonical markers for annotation for cell clusters; B. The difference in cellular proportion between RA and OA.

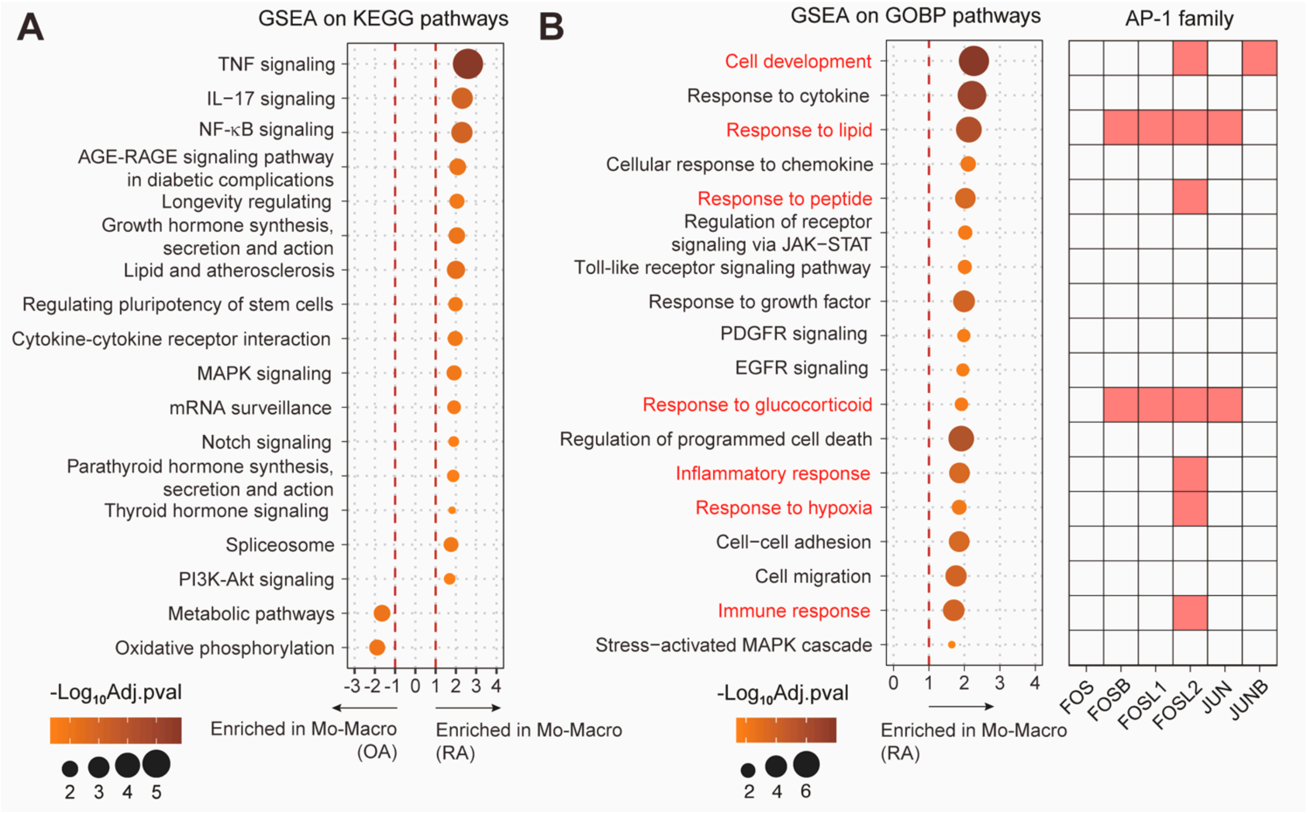

Figure S2. The gene set enrichment of KEGG and GOBP pathways in the comparison between RA and OA. A. The gene set enrichment on KEGG pathways; B. The gene set enrichment on GOBP pathways. Abbreviation: AGE, advanced glycation end products; RAGE, receptor of advanced glycation end products.

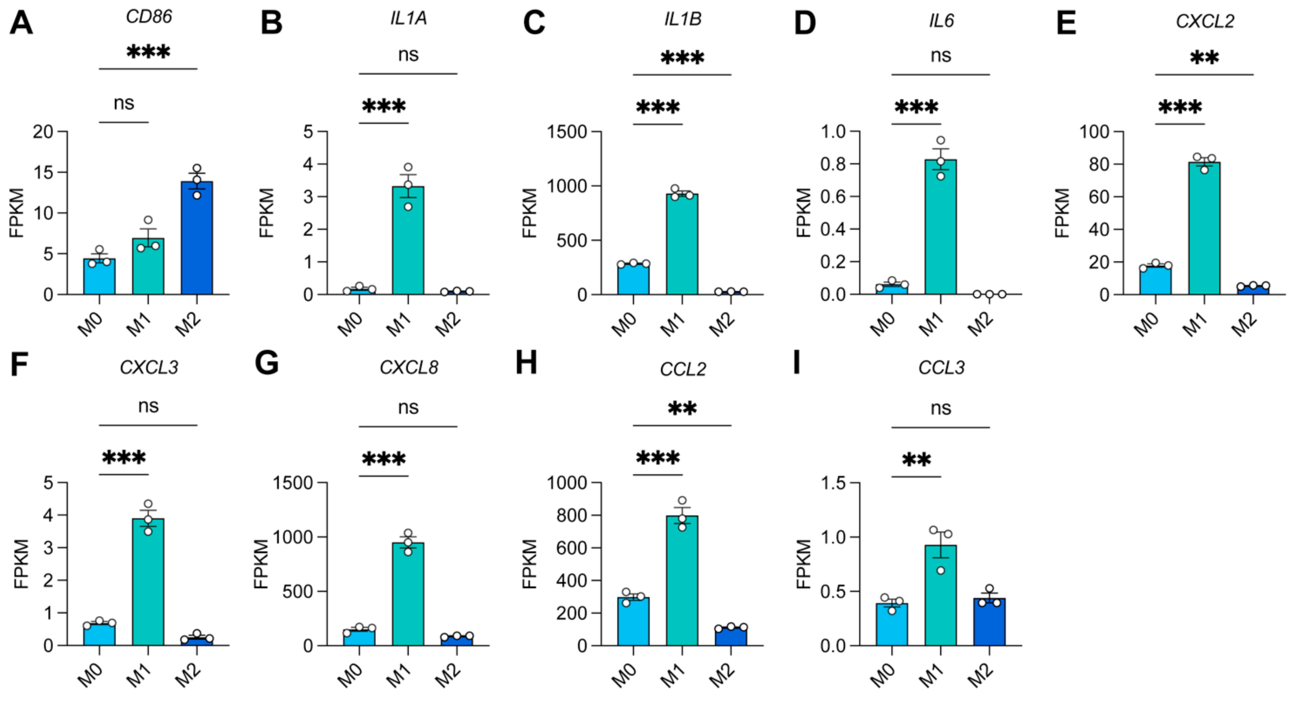

Figure S3. The comparisons of M1 marker expression among M0, M1, and M2 groups.

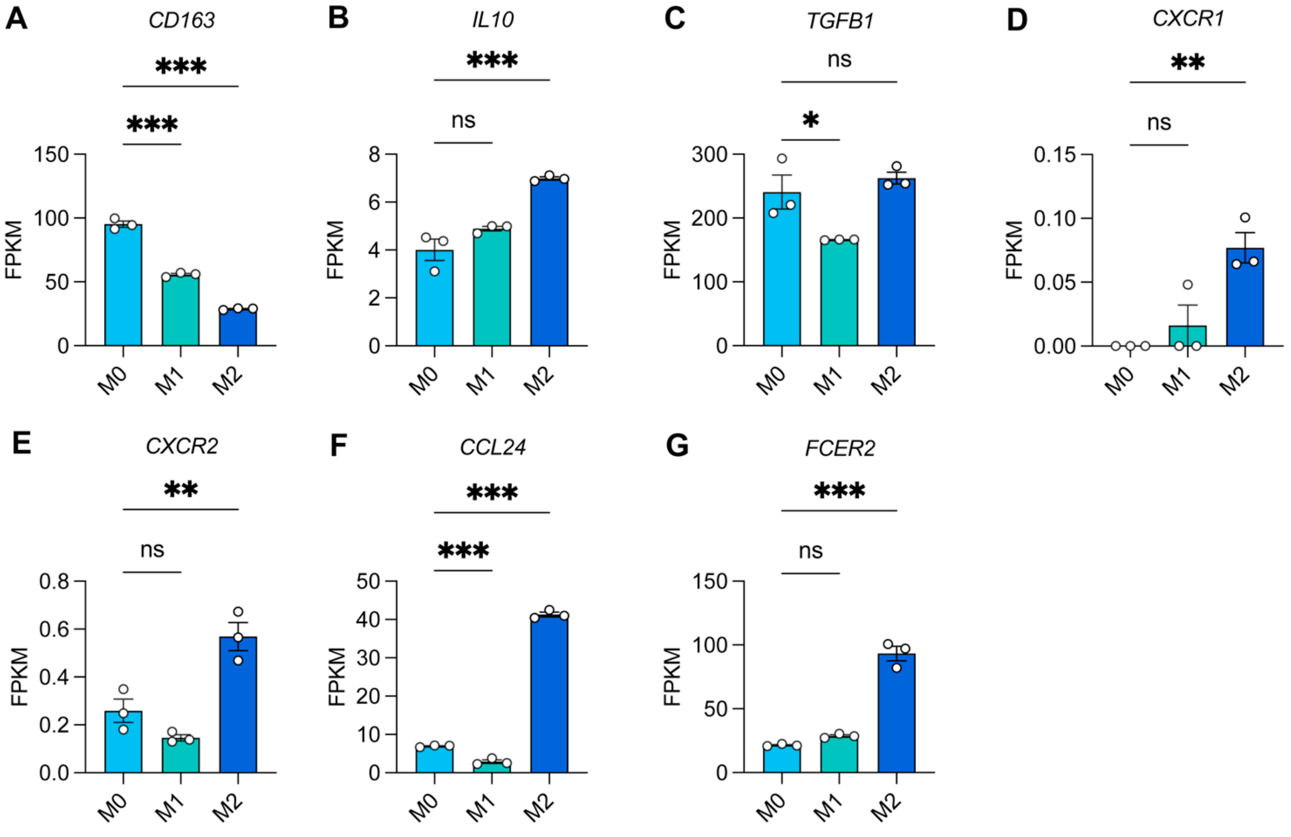

Figure S4. The comparisons of M2 marker expression among M0, M1, and M2 groups.

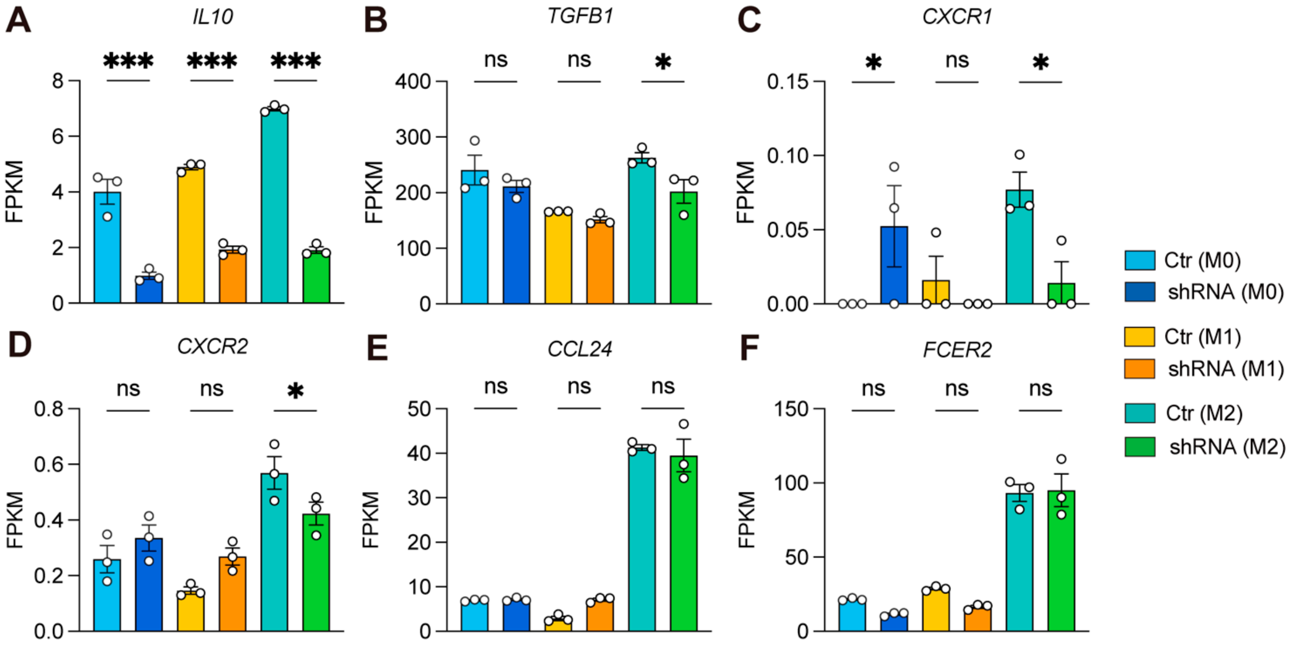

Figure S5. The comparisons of M2 markers after the knockdown of *FOSL2*.

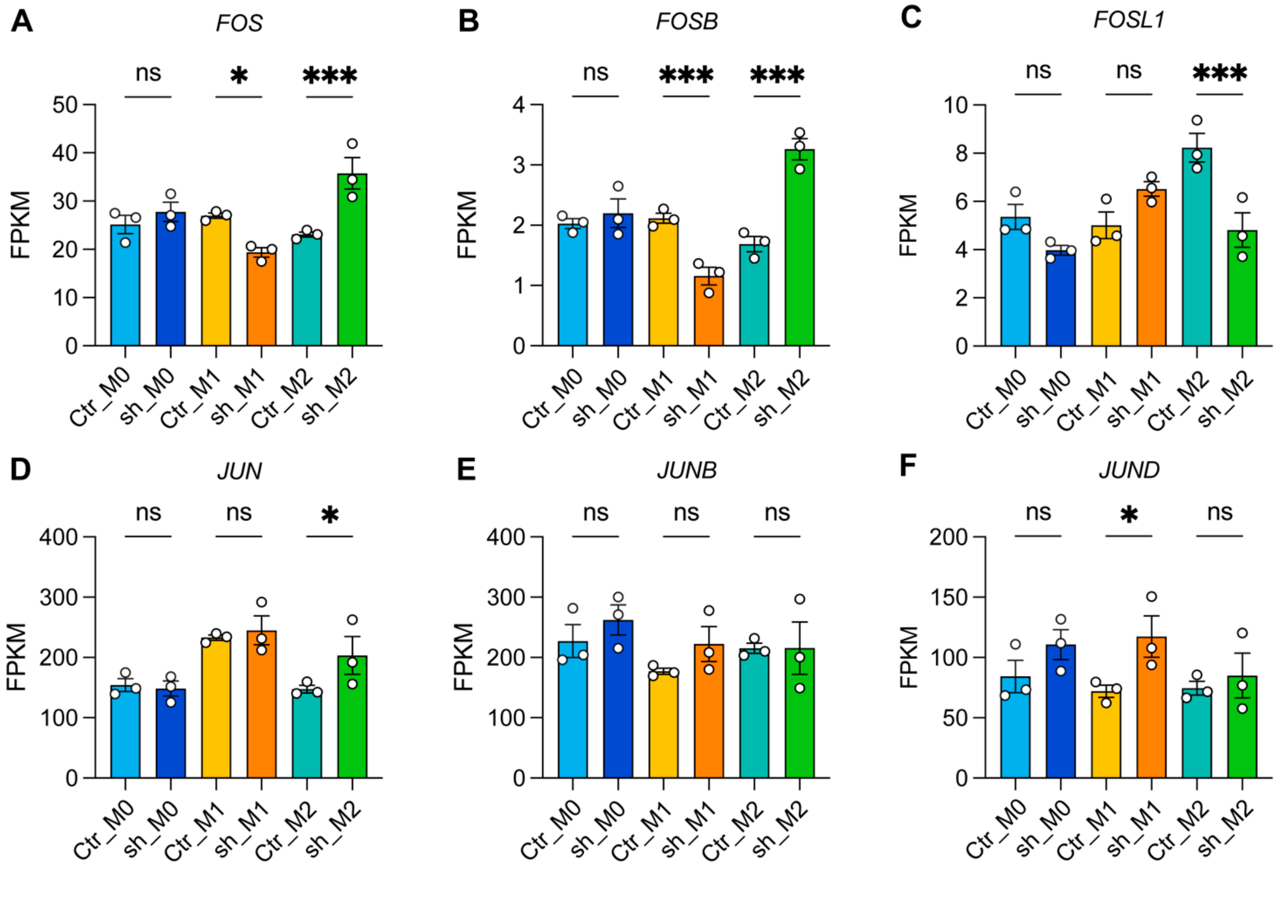

Figure S6. The expression of AP-1 members by M1/M2 cells after the knockdown of *FOSL2*.
