# Supplementary figures and images for: Synovial CXCL3+FOSL2+ Macrophages Mediate Inflammation via FOSL2/AP-1 in Rheumatoid Arthritis: A Single-Cell Transcriptome Analysis
Source: Int J Mol Sci. 2025 Oct 6;26(19):9718. doi: 10.3390/ijms26199718 (PMC12524658; doi:10.3390/ijms26199718)

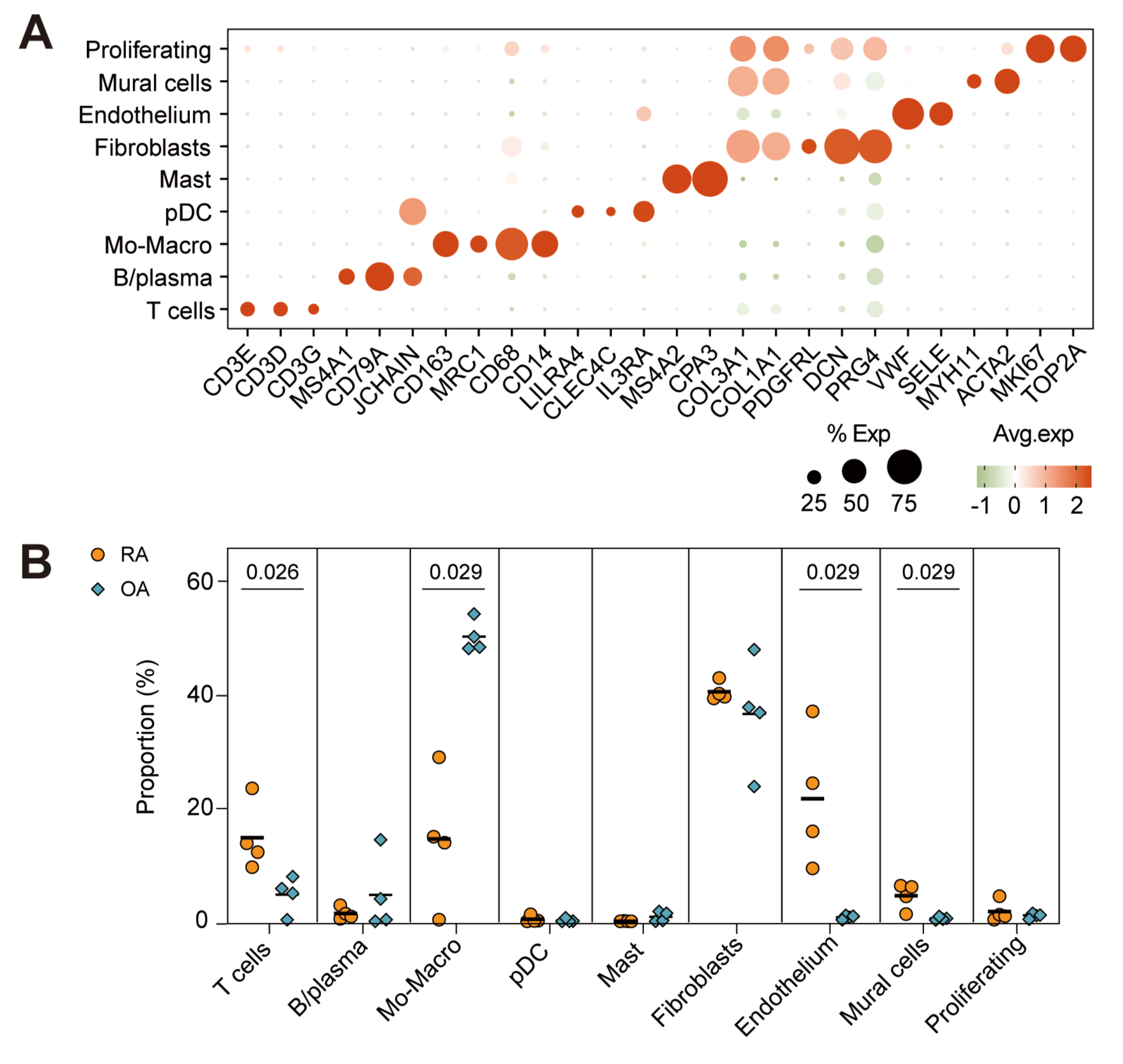

Supplement: Supplementary file 1 [file ijms-26-09718-s001.zip › Figure S1.tif]

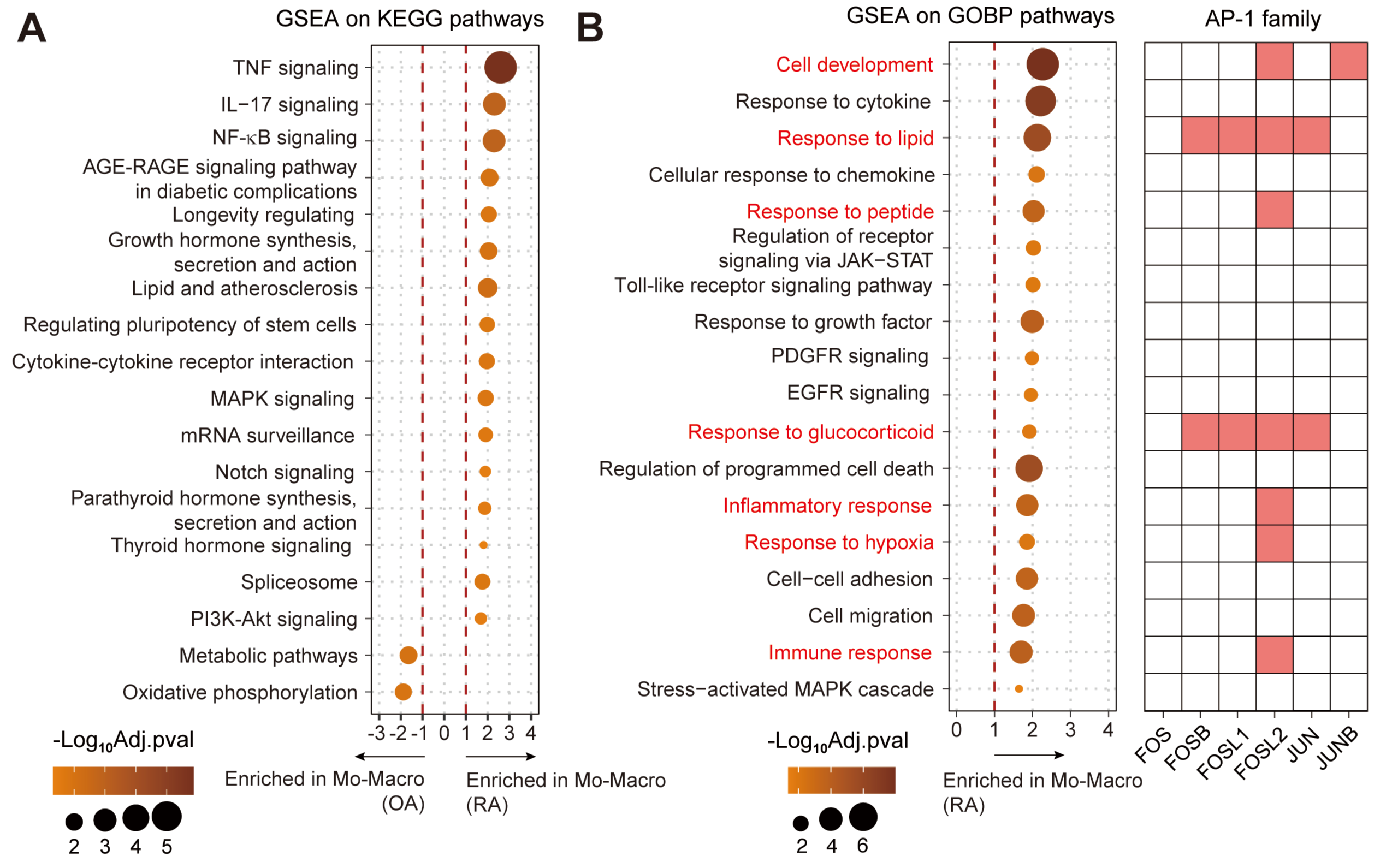

Supplement: Supplementary file 1 [file ijms-26-09718-s001.zip › Figure S2.tif]

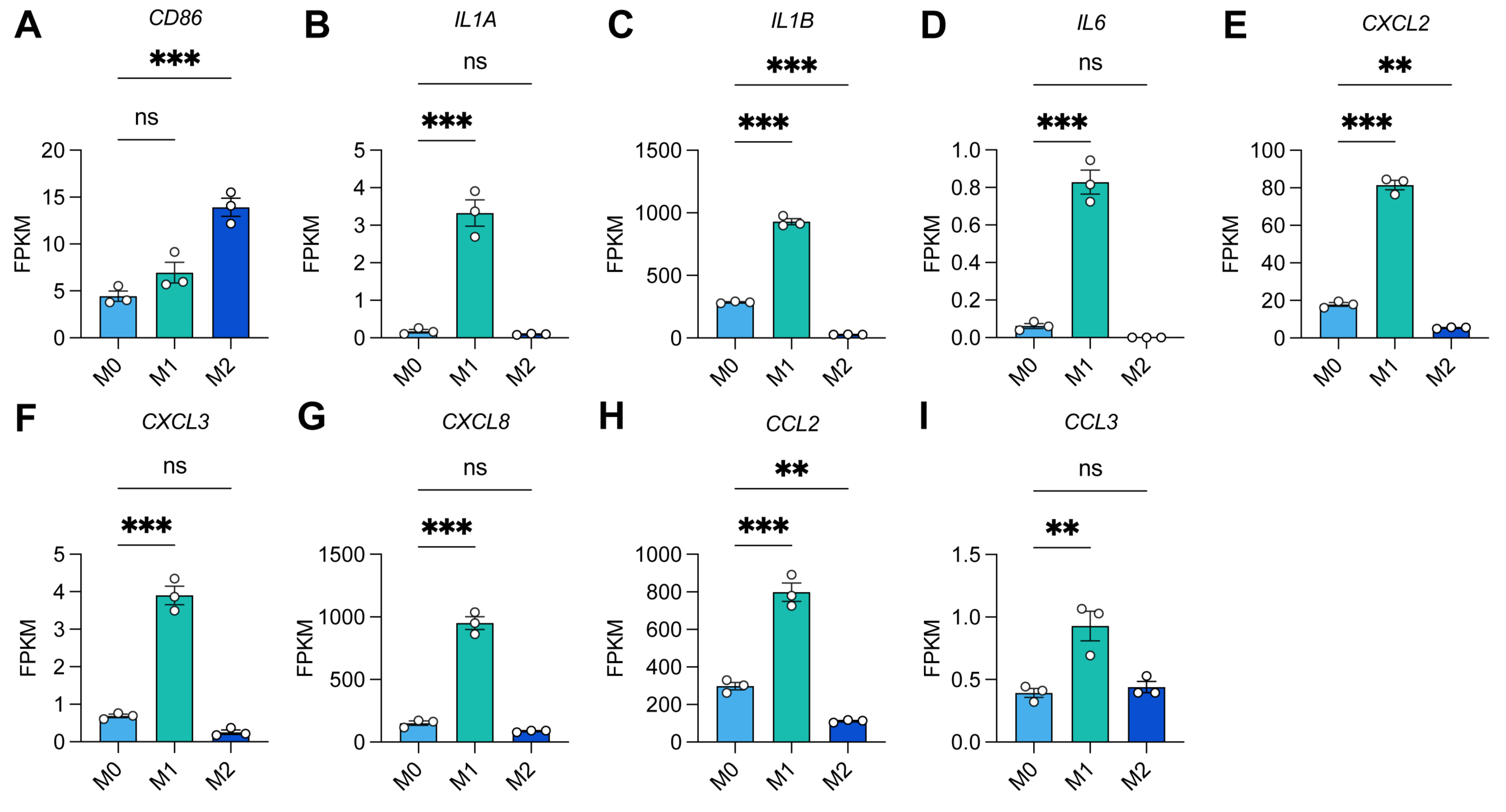

Supplement: Supplementary file 1 [file ijms-26-09718-s001.zip › Figure S3.tif]

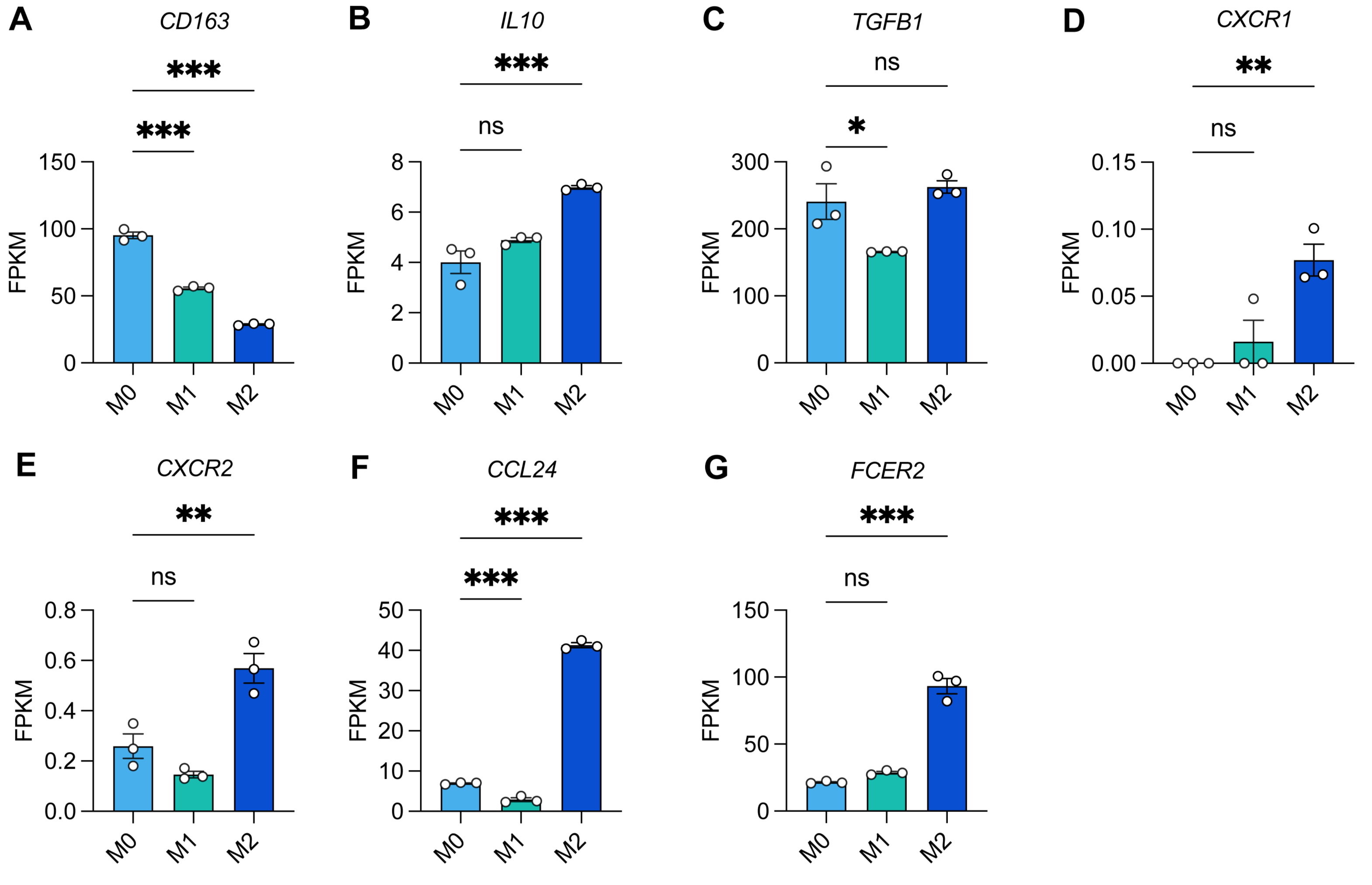

Supplement: Supplementary file 1 [file ijms-26-09718-s001.zip › Figure S4.tif]

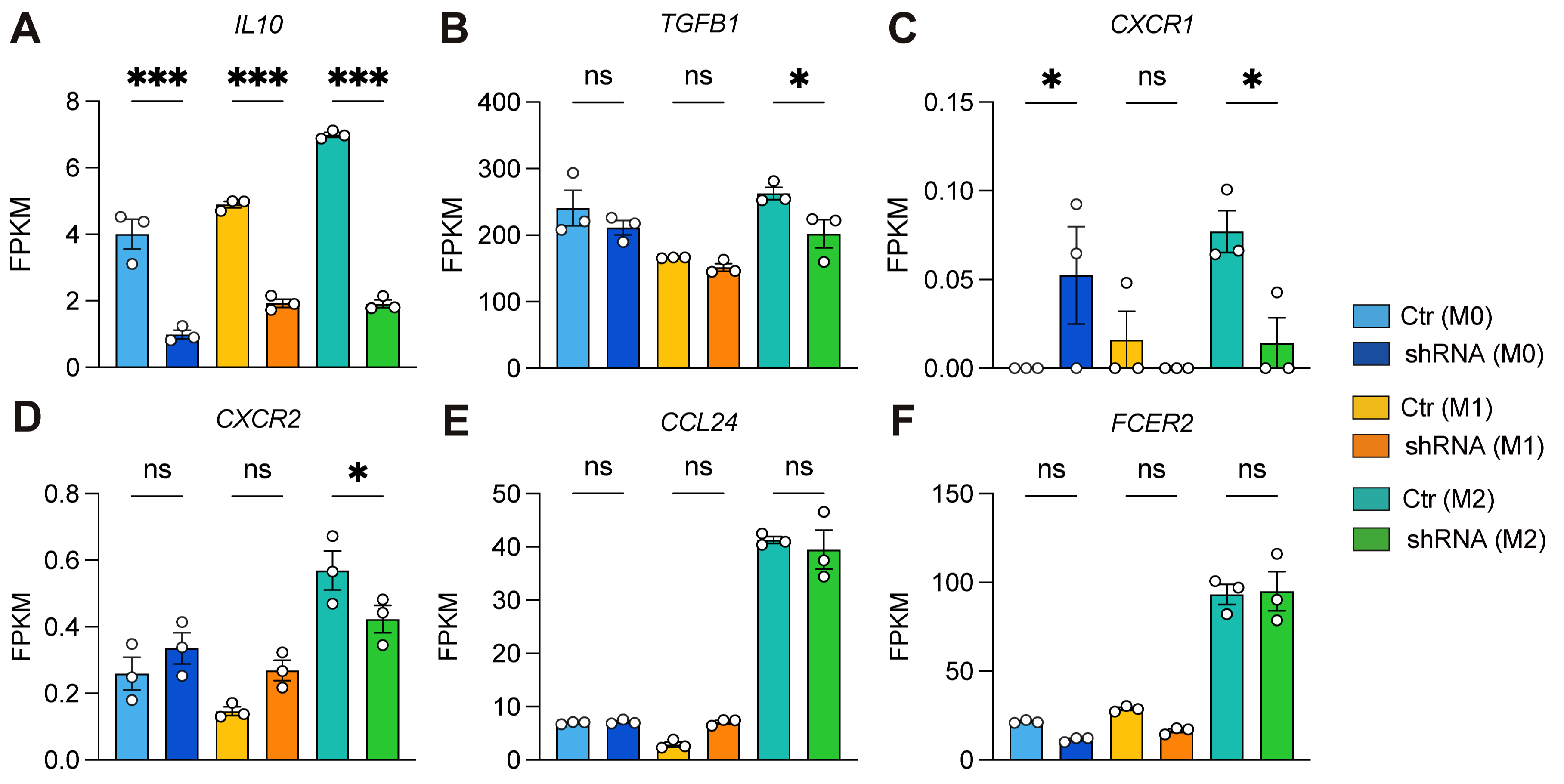

Supplement: Supplementary file 1 [file ijms-26-09718-s001.zip › Figure S5.tif]

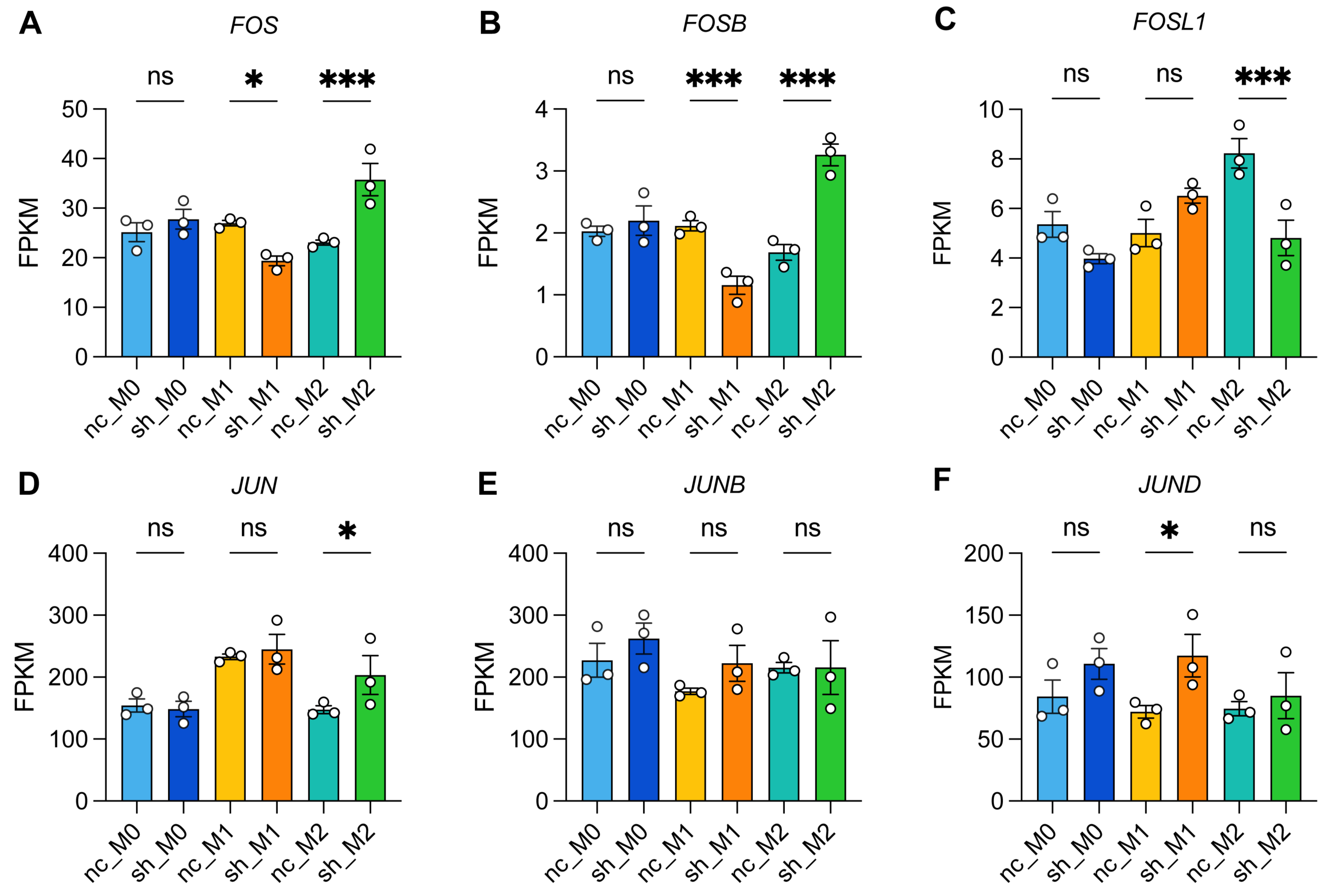

Supplement: Supplementary file 1 [file ijms-26-09718-s001.zip › Figure S6.tif]
